# Evolutionary game model of health care and social care collaborative services for the elderly population in China

**DOI:** 10.1186/s12877-022-03300-3

**Published:** 2022-07-25

**Authors:** Yin Sun, Xudong Zhang, Yuehong Han, Bo Yu, Haidan Liu

**Affiliations:** 1 Faculty of Management and Economics, Kunming University of Science and Technology, Kunming, Yunnan Province China; 2 School of Marxism, Kunming University of Science and Technology, Kunming, Yunnan Province China; 3 School of Humanities and Management, Yunnan University of Chinese Medicine, Kunming, Yunnan Province China

**Keywords:** Integrated care, Synergy, Evolutionary game

## Abstract

**Introduction:**

The synergy of health care and elderly social care organizations has become the focus of the research on integrated health care and social care. This study aims to propose a collaborative strategy among health care and elderly social care service providers.

**Methods:**

An evolutionary game model is applied for performance analysis and optimization of the cooperation between health care and elderly social care organizations. The behavioural strategies and the impact of key parameters on promoting the cooperation of the players are presented in detail.

**Results:**

Simulation experiments and sensitivity analysis results indicate that (1) the behavioural evolution of health care organizations and elderly social care organizations forms three types of integrated health care and social care services, namely, the bilateral cooperation type, health care organization-led type and elderly social care organization-led type. (2) Increasing the additional benefits for cooperation and reducing the additional costs for cooperation can promote the willingness to synergize to provide integrated health care and elderly social care services. At the early stage of evolution, increasing the costs that elderly social care organizations pay to purchase health care services or pay for negotiation in the bilateral cooperation type can provide incentives for health care organizations to cooperate while reducing the cooperation preferences of elderly social care organizations. However, the long-term impact of the costs on the behavioural strategies for cooperation of the two players cannot be determined.

**Conclusion:**

The behavioural decisions on cooperation between health care and elderly social care organizations influence each other; commitment to integration and effective collaboration can be achieved by increasing the additional benefits and reducing the marginal costs. The findings suggest that the political-economic context and government policies have a greater influence on promoting cooperation, thus yielding positive or negative results for integrated care practice.

## Introduction

China has the largest elderly population and is one of the most rapidly ageing countries in the world. The seventh national census shows that there are 264.02 million people aged over 60 live in China, among whom, 190.63 million are older than 65 years and 75% have non-communicable diseases, with estimates of more than 40 million with disabilities. Unfortunately, the poor coordination of health care and elderly social care services is perceived to be a significant barrier because the funding streams for health care and elderly social care are from the National Health Commission and Ministry of Civil Affairs, respectively. This raises the concern that the elderly population living at home or in long-term care facilities faces care gaps in geriatric medicine, rehabilitation, psychiatric, and palliative care services. Moreover, frail older people increasingly span the boundary between the two systems due to the financial burden [1]. The fragmented model of care delivery undermines efficient care to address older people’s complex needs; thus, health care for elderly people has generated widespread social concern. In this regard, various policies and strategies are currently underway to improve the collaborative governance of the provision and cost-effectiveness of care. Integrating health care and social care services and reforming the chronically underfunded social care system will undergo a profound change to address the demands of an ageing society in China [2].

This study aims to propose a collaborative strategy between health care and elderly social care service providers. An evolutionary game model based on synergy theory is introduced. Furthermore, on the basis of stability analysis and parameters, sufficient conditions are studied, and the practical barriers to the strategy choice towards an attractive solution in China are also described.

## Literature Review

In the 1970s, Western countries began to pay attention to the mechanisms by which health and local authorities provided joint services [3], particularly the fragmentation of social care responsibilities [4]. Since then, many countries have proposed integrated care for elderly individuals and explored their own patterns, for instance, integrated care in the U.K., the Program of All-Inclusive Care for the Elderly in America, and the long-time care insurance system in Japan. While there are numerous definitions of integrated care, a common understanding throughout is the ‘patient-centred’ aim of integrated care [5, 6]. Integration includes horizontal integration and vertical integration [7]. Horizontal integration means integration appearing between providers at the same level, such as the radial network formed by primary health care organizations and community elderly social care organizations. Vertical integration means the integration appearing between providers at different stages of care within the health system, which is called the supply chain or care pathway, for instance, two-way referral with shared processes between large general hospitals and rehabilitation centres or primary health care organizations. Integrated care holds the prospect of addressing the complex health and care needs of older people, especially patients with chronic multimorbidities [2, 8] and mental health conditions [9].

The policies and implementation effects of integrated care have become research hotspots. Glendinning evaluated two policy initiatives involving health care and social care organizations—between family doctors (general practitioners) and community health services and between health care and social care services organizations. In doing so, he found that structural integration could transform the decentralized system into service planning and delivery with a synergistic amplification effect [10]. The literature has shown that the integration of health care and social care can enhance health outcomes [11], achieve quality, efficiency and cost control [7, 9], and is expected to reduce overtreatment, waste and redundancy [12]. A review served as a reminder that the development and push to achieve a greater integration of services may have some benefits for organizations, users and carers of services, but more work must be done to realize the full potential of the new policy agenda on integration [3]. Some different views have been proposed. Studies have revealed that it is difficult to prove that integrated care can reduce direct or indirect costs [13, 14] or improve cost effectiveness [15]. Although it is conducive to improving service accessibility if shifting care to the community, there is little evidence demonstrating a sustained reduction in hospital use [16]. One possible reason is that the elements from integrated care schedules are not always appropriately or fully implemented, which results in different practical results [17–19]. This means policy-makers cannot ignore the potential benefits of integrated care; however, more rigorous research should be conducted and a better framework should be designed. It is widely agreed that if synergies exist and organizations are willing to collaborate, patient care improves. This, however, has been demonstrated to be not the case [20]. Some pioneers thought it might take five years or longer to deliver interventions to show demonstrable impacts [20]. T. Chen et al. constructed a utility model of resource allocation under the premise of the maximization of elderly social care organizations and health care organizations’ own benefits to explore how health care organizations and elderly social care organizations invest resources during integration [21]. Researchers have identified numerous challenges in, factors of, elements of, barriers to and facilitators of successful integrated care [3, 22, 23]. Holterman et al. concluded that enabling policy and funding played an important role in the sustained implementation of integrated care services [24]. Maruthappu et al. stressed some core factors, such as adequate financing, cultural change and supportive regulation, to accomplish integration [7]. Research has indicated the importance of collaborative governance [25] and hierarchical governance [26] under the circumstances of continuously evolving policy environments. Incomplete information (for example, due to the difficulty of linking health and social care data) and the differences in culture and governance may fail to provide accessible and appropriate services to older people [16]. At the level of the environment and system, the elements of community and social resources (i.e., in the form of community health teams or home care services) and support (i.e., from family caregivers) of a person with multiple morbidities and the factors related to the financing component (such as the cost effectiveness of care and the financial incentives or reimbursement systems) are often marginalized [23].

Integrated health care and social care services require segmentation, integration, transformation and upgrading of traditional health care resources and elderly social care resources. The differences in resource types, quantity and quality between health care organizations and elderly social care organizations are not only the motivation for cooperation but also the source of conflict [27]. Health care fields involve high technicality, high professionalism and high barriers to entry. In addition, China is still short of high-quality health care resources. Health care organizations, especially large and medium-sized health care organizations, often occupy a dominant position when participating in integrated health care and social care services, on which elderly social care organizations have asymmetric resource dependence [28], resulting in unequal power between them [29] and even conflicts. In addition, at the early development stage of integrated health care and social care, limited by resources and imperfect systems and policies, the decisions of health care organizations and elderly social care organizations often conflict between individual rationality and collective rationality to strive for subsidies and maximize their own interests [30]. The competition caused by these conflicts, if not intervened in, will put the system in a disordered state.

Synergetics is a subject that studies ordered and self-organized collective behaviour under the control of universal laws. The concept of “synergy” was first proposed by Hermann Haken in 1971, and then, he systematically expounded synergy theory. Synergetics mainly studies the synergistic effects between open complex systems composed of a large number of subsystems through energy or material exchange with the outside world and the interaction of internal subsystems as well as the mechanism from chaos to order macroscopically, and it reveals the common law of the evolution of complex systems to macro-order structure. The key to the transformation of a complex system into an ordered state is the self-organization process among subsystems, which generates a new ordered structure, and the cooperation and competition within the complex system are the driving forces of subsystem self-organization evolution [31–33]. In view of synergy theory, when chaos produces order or one order changes into another order, recognizing the internal self-organization mechanism of the system and applying synergy theory to formulate corresponding policies will achieve greater benefits through less investment [34]. Therefore, synergy theory has been applied to explore how to construct an old-age service system in recent years. Existing studies mainly focus on the synergy mechanism of service providers [35], synergetic governance between the government and other subjects [36], the coordination in regional elderly social care service development [37] and the coordination between ageing undertakings and ageing industrial development [38].

Axelrod explored the conditions under which cooperation would emerge in a world of egoists without central authority and showed that the evolutionary approach was the most general since all possible strategies can be taken into account to achieve cooperation under anarchy [39]. Axelrod further indicated the necessary and sufficient conditions for a strategy to be collectively stable [40]. Since then, the evolutionary approach has been widely used in the fields of political philosophy and economic and social exchanges, and it has played an important role. In the traditional prisoner’s dilemma game model, it is assumed that the players are equal, and cooperation or defection brings the same outcomes for both subjects. In fact, integration occurs between organizations that are unequal in terms of (political) influence or resources. In this situation, the outcomes brought by strategy selection are obviously different. Therefore, the inequality between two subjects and the impact of government regulation are taken into account when running the game model.

There is a lack of synergy among integrated health care and social care service providers in China [30, 41], and the lack of synergy between health and other social welfare services will reduce the efficiency and effectiveness with which patients’ complex problems are solved [4]. How to achieve synergy between health care organizations and elderly social care organizations is one of the sticking points. Existing studies emphasize the importance of integrated health care and social care but mainly focus on the theoretical argumentation of supporting policies, the evaluation of implementation and promotion or obstacles. There is little research on strategies selection and synergetic behavioural evolution between health care organizations and elderly social care organizations.

In China, health care organizations/elderly social care organizations have to provide extra elderly social care services/health care services under policy pressures and central control. For the purpose of maximizing individuals’ benefit, they often have little enthusiasm for providing integrated health care and social care services for elderly individuals, resulting in inadequate policy implementation and an incomplete integration of health care and elderly social care services. This situation is challenging when considering Axelrod’s suggestion that mutual cooperation can emerge in a world of egoists without central control by starting with a cluster of individuals who rely on reciprocity [40]. Thus, it is worth seeing whether health care organizations and elderly social care organizations are willing to synergize with each other to improve their connectivity mainly depending on the trade-off between various “payments” and “benefits”. In the Chinese context, the parameters are further optimized when constructing an evolutionary game model in this study to test organizations’ cooperation strategies among service providers and to identify the key intervention points in decision-making. The application of an evolutionary game model contributes to helping us grasp synergistic evolutionary behaviours, which is of great significance for improving service efficiency and achieving the deep integration of health care and social care services.

We contribute to the literature in the following main ways: (1) We run an evolutionary game model from the perspective of subject synergy between elderly social care organizations and health care organizations to study the factors and evolutionary path of their decision-making. (2) We combine numerical simulation with practice and identify the obstacles restricting the synergy between health care organizations and elderly social care organizations, which breaks through the previous analysis mode of single numerical simulation. (3) We consider the inequality of service providers and the impact of government regulation on strategies when setting parameters.

## Methods

### Design and Basic Assumptions

In the traditional cooperative game model, it is usually assumed that both players have unbounded rationality and that the game players are equal. In the process of providing integrated health care and social care services, the main players of the game are health care organizations and elderly social care organizations. It is assumed that they are both bounded rationally and choose strategies independently and dynamically based on returns. Moreover, the players are unequal in terms of (political) influence or resources. Clearly, the outcomes brought by strategy selection are different.

There are three main forms of integrated health care and social care services in China: the health care organization-led type, the elderly social care organization-led type and the bilateral cooperation type. The “participation” strategy of elderly social care organizations refers to providing health care service, in addition to elderly social care services. In this case, if health care organizations also choose the “participation” strategy, the form of integrated health care and social care services is bilateral cooperation. If health care organizations choose the “nonparticipation” strategy, elderly social care organizations have to set up their own health care organization to provide integrated health and care services, and the form of integrated health care and social care services is dominated by elderly social care organizations. The “nonparticipation” strategy of elderly social care organizations means that they do not provide health care services. In this case, if health care organizations choose the “participation” strategy, health care organizations should provide extra elderly social care services, and the form of integrated health and care services is the health care organization-led type. If health care organizations choose the “nonparticipation” strategy, they do not provide extra services, and integrated health care and social care services cannot be provided.

In the game model, the game strategy combination of elderly social care organizations is $\left\{\mathrm{participation},\mathrm{ nonparticipation}\right\}$. The probability of elderly social care organizations choosing the participation strategy is $\left(0\le x\le 1\right)$, and the probability of choosing nonparticipation is $\left(1-x\right)$. Similarly, the health care organizations’ game strategy combination is $\left\{\mathrm{participation},\mathrm{ nonparticipation}\right\}$. The probability of health care organizations choosing the participation strategy is $\left(0\le y\le 1\right)$, and the probability of choosing nonparticipation is $\left(1-y\right)$.

The parameter definitions and assumptions are as follows. (1) When elderly social care organizations provide extra health care services, if they establish a cooperative relationship with health care organization, the additional benefit of elderly social care organizations from the provision of health care services is ${U}_{1}$, and the costs that elderly social care organizations pay to purchase health care services or pay for negotiation are ${P}_{1}$. The government subsidy to elderly social care organizations is ${S}_{1}$. If elderly social care organizations do not cooperate with health care organizations, the additional benefit of elderly social care organizations is ${U}_{2}$ $\left({U}_{2}>{U}_{1}\right)$, and the cost for elderly social care organizations providing extra health care services is ${P}_{2}$ $\left({P}_{2}>{P}_{1}\right)$. In this situation, the government subsidy to elderly social care organizations is ${S}_{2}$ $\left({S}_{2}>{S}_{1}\right)$. (2) When health care organizations provide extra elderly social care services, if they cooperate with elderly social care organizations, the additional income of health care organizations from the provision of elderly social care services is ${R}_{1}$, and the additional operating cost is ${C}_{1}$. If health care organization do not cooperate with elderly social care organizations, the additional income of health care organizations from the extra provision of elderly social care services is ${R}_{2}$ $\left({R}_{2}>{R}_{1}\right)$, and the extra cost is ${C}_{2}$ $\left({C}_{2}>{C}_{1}\right)$. The government subsidy to health care organizations is ${S}_{1}^{^{\prime}}$. The relevant parameters and their explanations are shown in Table 1.

**Table 1:** Related parameters and their explanation

Symbol	Explanation
${U}_{1}$	The additional benefit of elderly social care organizations from the provision of health care services in the bilateral cooperation type
${U}_{2}$	The additional benefit of elderly social care organizations in the elderly social care organization-led type
${P}_{1}$	The costs that elderly social care organizations pay to purchase health care services or pay for negotiation in the bilateral cooperation type
${P}_{2}$	The cost for elderly social care organizations providing extra health care services in the elderly social care organization-led type
${R}_{1}$	The additional income of health care organizations from the provision of elderly social care services in the bilateral cooperation type
${R}_{2}$	The additional income of health care organizations from the extra provision of elderly social care services in the health care organization-led type
${C}_{1}$	The additional operating costs of health care organizations in the bilateral cooperation type
${C}_{2}$	The extra costs of health care organizations from the extra provision of elderly care services in the health care organization-led type
${S}_{1}$	The government subsidy to elderly social care organizations in the bilateral cooperation type
${S}_{2}$	The government subsidy to elderly social care organizations in the elderly social care organization-led type
${S}_{1}^{^{\prime}}$	The government subsidy to health care organizations in the health care organization-led type

There are several kinds of benefits from cooperation, such as the benefit from increased occupancy due to providing integrated health care and social care services [42], the service income of health care organizations due to the additional provision of elderly social care service or the service income of elderly social care organizations due to the additional provision of health care service, the benefit from the increase in sick patients in health care organizations and the benefit from the increase in rehabilitation patients in elderly social care organizations due to mutual referral, the savings in operating and purchasing equipment due to resource sharing [43], and the increase in the social benefit due to the provision of more comprehensive services. There are also some kinds of costs from cooperation, such as the investment of manpower, material resources, capital and personnel training, the increased cost due to the adjustment of health care insurance settlement and government subsidy policies, and the increased regulatory cost due to cooperation [43].

### Model Construction

Based on the above assumptions, combined with the costs, benefits and losses of health care organizations and elderly social care organizations, we construct an evolutionary game income matrix between health care organizations and elderly social care organizations, as shown in Table 2.

**Table 2:** The evolutionary game income matrix between health care organizations and elderly social care organizations

		Health care organizations	
		Participation $\left(y\right)$	Nonparticipation $\left(1-y\right)$
Elderly social care organizations	Participation $\left(x\right)$	${U}_{1}+{S}_{1}-{P}_{1}$	${U}_{2}+{S}_{2}-{P}_{2}$
		${R}_{1}+{P}_{1}-{C}_{1}$	$0$
	Nonparticipation $\left(1-x\right)$	0	0
		${R}_{2}+{S}_{1}^{^{\prime}}-{C}_{2}$	$0$

### Model Derivation

The expected benefit of elderly social care organizations’ choice of participation and nonparticipation is ${E}_{11}$ and ${E}_{12}$, respectively, and the average expected benefit of elderly social care organizations is ${\overline{E} }_{1}$.

1 $${E}_{11}=y\left({U}_{1}+{S}_{1}-{P}_{1}\right)+\left(1-y\right)\left({U}_{2}+{S}_{2}-{P}_{2}\right)$$

2 $${E}_{12}=0$$

3 $${\overline{E} }_{1}=x{E}_{11}+\left(1-x\right){E}_{12}$$

The expected benefit of health care organizations choosing participation and nonparticipation is ${E}_{21}$ and ${E}_{22}$, respectively, and the average expected benefit of health care organizations is ${\overline{E} }_{2}$.

4 $${E}_{21}=x\left({R}_{1}+{P}_{1}-{C}_{1}\right)+\left(1-x\right)\left({R}_{2}+{S}_{1}^{^{\prime}}-{C}_{2}\right)$$

5 $${E}_{22}=0$$

6 $${\overline{E} }_{2}=y{E}_{21}+\left(1-y\right){E}_{22}$$

Based on the Malthusian equation, the replication dynamic equation of proportion $x$ for elderly social care organizations can be deduced from Eqs. (1) , (2) and  (3), and it is shown in Eq. (7):

$$F\left(x\right)=\frac{\mathrm{d}x}{\mathrm{d}t}=x\left({E}_{11}-{\overline{E} }_{1}\right)= x\left(1-x\right)\left({E}_{11}-{E}_{12}\right)$$

7 $$= x\left(1-x\right)\left[y\left({{U}_{1}-{U}_{2}+P}_{2}-{P}_{1}+{S}_{1}-{S}_{2}\right)+{U}_{2}+{S}_{2}-{P}_{2}\right]$$

Similarly, the replication dynamic equation of proportion $y$ for health care organizations can be deduced from Eqs. (4), (5) and  (6), and it is shown in Eq. (8):

$$F\left(y\right)=\frac{\mathrm{d}y}{\mathrm{d}t}=y\left({E}_{21}-{\overline{E} }_{2}\right)= y\left(1-y\right)\left({E}_{21}-{E}_{22}\right)$$

8 $$= y\left(1-y\right)\left[x\left({R}_{1}-{R}_{2}+{P}_{1}-{S}_{1}^{^{\prime}}+{C}_{2}-{C}_{1}\right)+{R}_{2}+{S}_{1}^{^{\prime}}-{C}_{2}\right]$$

According to the stability theorem of differential equations, if a strategy is stable, the proportions $x$ and $y$ should meet the following conditions:

9 $$F\left(x\right)=0, \frac{\partial F\left(x\right)}{\partial x}<0$$

$$F\left(y\right)=0, \frac{\partial F\left(y\right)}{\partial y}<0$$

Let $F\left(x\right)=0$, $F\left(y\right)=0$; we obtain five local equilibrium points, which are $O\left(0, 0\right)$, $A\left(0, 1\right)$, $B\left(1, 0\right)$, $C\left(1, 1\right)$, and $E\left({x}^{*}, {y}^{*}\right)$, where ${x}^{*}=\frac{{C}_{2}-{R}_{2}-{S}_{1}^{^{\prime}}}{{R}_{1}-{R}_{2}+{P}_{1}-{S}_{1}^{^{\prime}}+{C}_{2}-{C}_{1}}$ and ${y}^{*}=\frac{{P}_{2}-\left({U}_{2}+{S}_{2}\right)}{{U}_{1}-{U}_{2}+{P}_{2}-{P}_{1}+{S}_{1}-{S}_{2}}$.

### Stability Analysis

Friedman [44] proposed that the evolutionary stable strategy (ESS) can be obtained through the Jacobian matrix $J$ computational analysis of the system. That is, if and only if determinant $J$ ($det J$) > 0 and trace $J$ ($tr J$) < 0, the point has local stability. The Jacobian matrix $J$ is shown in Eq. (10):

10 $$J=\left[\begin{array}{cc}\frac{\partial F\left(x\right)}{\partial x}& \frac{\partial F\left(x\right)}{\partial y}\\ \frac{\partial F\left(y\right)}{\partial x}& \frac{\partial F\left(y\right)}{\partial y}\end{array}\right]=\left[\begin{array}{cc}\left(1-2x\right)\left[y\left({{U}_{1}-{U}_{2}+P}_{2}-{P}_{1}+{S}_{1}-{S}_{2}\right)+{U}_{2}+{S}_{2}-{P}_{2}\right]& x\left(1-x\right)\left({{U}_{1}-{U}_{2}+P}_{2}-{P}_{1}+{S}_{1}-{S}_{2}\right)\\ y\left(1-y\right)\left({R}_{1}-{R}_{2}+{P}_{1}-{S}_{1}^{^{\prime}}+{C}_{2}-{C}_{1}\right)& (1-2y)\left[x\left({R}_{1}-{R}_{2}+{P}_{1}-{S}_{1}^{^{\prime}}+{C}_{2}-{C}_{1}\right)+{R}_{2}+{S}_{1}^{^{\prime}}-{C}_{2}\right]\end{array}\right]$$

The Determinant $J$ is as follows:

11 $$det J=\left(1-2x\right)\left[y\left({{U}_{1}-{U}_{2}+P}_{2}-{P}_{1}+{S}_{1}-{S}_{2}\right)+{U}_{2}+{S}_{2}-{P}_{2}\right]\left(1-2y\right)\left[x\left({R}_{1}-{R}_{2}+{P}_{1}-{S}_{1}^{^{\prime}}+{C}_{2}-{C}_{1}\right)+{R}_{2}+{S}_{1}^{^{\prime}}-{C}_{2}\right]-x\left(1-x\right)\left({{U}_{1}-{U}_{2}+P}_{2}-{P}_{1}+{S}_{1}-{S}_{2}\right)y\left(1-y\right)\left({R}_{1}-{R}_{2}+{P}_{1}-{S}_{1}^{^{\prime}}+{C}_{2}-{C}_{1}\right)$$

The Trace $J$ is as follows:

12 $$tr J=\left(1-2x\right)\left[y\left({{U}_{1}-{U}_{2}+P}_{2}-{P}_{1}+{S}_{1}-{S}_{2}\right)+{U}_{2}+{S}_{2}-{P}_{2}\right]+\left(1-2y\right)\left[x\left({R}_{1}-{R}_{2}+{P}_{1}-{S}_{1}^{^{\prime}}+{C}_{2}-{C}_{1}\right)+{R}_{2}+{S}_{1}^{^{\prime}}-{C}_{2}\right]$$

The Determinant and Trace of the Jacobian matrix at five local equilibrium points are shown in Table 3.

**Table 3:** The Determinant and Trace at each equilibrium point

Equilibrium point	Determinant	Trace
$O\left(0, 0\right)$	$\left({U}_{2}+{S}_{2}-{P}_{2}\right)\left({R}_{2}+{S}_{1}^{^{\prime}}-{C}_{2}\right)$	${U}_{2}+{S}_{2}-{P}_{2}+{R}_{2}+{S}_{1}^{^{\prime}}-{C}_{2}$
$A\left(0, 1\right)$	$\left({U}_{1}+{S}_{1}-{P}_{1}\right)\left({C}_{2}-{R}_{2}-{S}_{1}^{^{\prime}}\right)$	${U}_{1}+{S}_{1}-{P}_{1}+{C}_{2}-{R}_{2}-{S}_{1}^{^{\prime}}$
$B\left(1, 0\right)$	$\left({P}_{2}-{U}_{2}-{S}_{2}\right)\left({R}_{1}+{P}_{1}-{C}_{1}\right)$	${P}_{2}-{U}_{2}-{S}_{2}+{R}_{1}+{P}_{1}-{C}_{1}$
$C \left(1, 1\right)$	$\left({U}_{1}+{S}_{1}-{P}_{1}\right)\left({R}_{1}+{P}_{1}-{C}_{1}\right)$	${C}_{1}-{R}_{1}-{U}_{1}-{S}_{1}$
$E\left({x}^{*}, {y}^{*}\right)$	$-\frac{\left({P}_{2}-{U}_{2}-{S}_{2}\right)\left({C}_{2}-{R}_{2}-{S}_{1}^{^{\prime}}\right)\left({U}_{1}+{S}_{1}-{P}_{1}\right)\left({R}_{1}+{P}_{1}-{C}_{1}\right)}{\left({{U}_{1}-{U}_{2}+P}_{2}-{P}_{1}+{S}_{1}-{S}_{2}\right)\left({R}_{1}-{R}_{2}+{P}_{1}-{S}_{1}^{^{\prime}}+{C}_{2}-{C}_{1}\right)}$	0

If $det J>0$ and $tr J<0$ are satisfied at the same time, the local equilibrium point is the evolutionary stable equilibrium point of the system [45].

1. When ${U}_{1}+{S}_{1}<{P}_{1}$ and ${R}_{2}+{S}_{1}^{^{\prime}}>{C}_{2}$, $A\left(0, 1\right)$ is the evolutionary stable equilibrium point. For elderly social care organizations, the sum of additional benefits and government subsidies is less than the costs that elderly social care organizations pay to purchase health care services or pay for negotiation. For health care organizations, the additional benefits and government subsidies for self-operated elderly care beds are more than the additional operating costs. In this case, the ESS is that health care organizations provide extra elderly social care services by themselves, and the type of integrated health care and social care is the health care organization-led type.
2. When ${U}_{2}+{S}_{2}>{P}_{2}$ and ${R}_{1}+{P}_{1}<{C}_{1}$, $B\left(1, 0\right)$ is the evolutionary stable equilibrium point. For elderly social care organizations, the sum of additional benefits from providing health care services and government subsidies is more than the costs for elderly social care organization to provide health care services. For health care organizations, the additional benefits from cooperation with elderly social care organizations and the costs that elderly social care organizations pay to purchase health care services or pay for negotiation are less than the extra operating costs. In this case, the ESS is that elderly social care organizations set up health care organizations to provide health care services, and the type of integrated health care and social care is the elderly social care organization-led type.
3. When ${U}_{1}+{S}_{1}>{P}_{1}$ and ${R}_{1}+{P}_{1}>{C}_{1}$, $C\left(1, 1\right)$ is the evolutionary stable equilibrium point. For elderly social care organizations, the sum of additional benefits and government subsidies is more than the costs that elderly social care organizations pay to purchase health care services or pay for negotiation. For health care organizations, the additional benefits from cooperation with elderly social care organizations and the costs that elderly social care organizations pay to purchase health care services or pay for negotiation are more than the extra operating costs. At that time, the ESS is that health care organizations and elderly social care organizations cooperate to provide integrated health care and social care services, and the type of integrated health care and social care is bilateral cooperation.

This paper focuses on promoting the subject synergy of integrated health care and social care services for elderly people, so it mainly analyses the stability of each local equilibrium point only when $C\left(1, 1\right)$ is the ESS. When ${U}_{1}+{S}_{1}>{P}_{1}$ and ${R}_{1}+{P}_{1}>{C}_{1}$, the stability analysis of each equilibrium point can be divided into 4 scenarios, and the stability analysis results are shown in Table 4.

**Table 4:** Local stability analysis results in each scenario

Equilibrium point	Scenario 1			Scenario 2			Scenario 3			Scenario 4		
	$det J$	$tr J$	Result	$det J$	$tr J$	Result	$det J$	$tr J$	Result	$det J$	$tr J$	Result
$O\left(0, 0\right)$	$+$	$-$	ESS	$-$	$\pm$	Saddle	$-$	$\pm$	Saddle	$+$	$+$	Unstable
$A\left(0, 1\right)$	$+$	$+$	Unstable	$-$	$\pm$	Saddle	$+$	$+$	Unstable	$-$	$\pm$	Saddle
$B\left(1, 0\right)$	$+$	$+$	Unstable	$+$	$+$	Unstable	$-$	$\pm$	Saddle	$-$	$\pm$	Saddle
$C \left(1, 1\right)$	$+$	$-$	ESS	$+$	$-$	ESS	$+$	$-$	ESS	$+$	$-$	ESS
$E\left({x}^{*}, {y}^{*}\right)$	$-$	0	Saddle			None			None			None

**Fig. 1:**
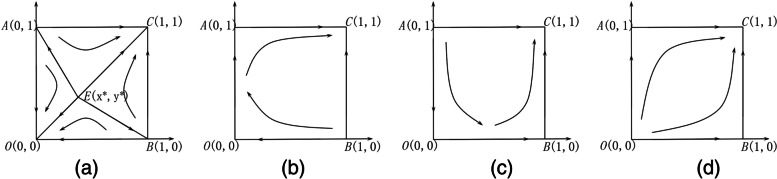
The evolution phase diagrams of the system in each scenario: (**a**) scenario 1; (**b**) scenario 2; (**c**) scenario 3; (**d**) scenario 4

Scenario 1: When ${U}_{2}+{S}_{2}<{P}_{2}$ and ${R}_{2}+{S}_{1}^{^{\prime}}<{C}_{2}$, this means that, in the elderly social care organization-led type, the additional benefits of elderly social care organizations are less than the costs for elderly social care organizations to provide extra health care services. In the health care organization-led type, health care organizations provide extra elderly social care services, and the additional benefits and government subsidies for self-operated elderly care beds are less than the additional operating costs. The evolutionary phase diagram is shown in Fig. 1(a). There are two evolutionary stable strategies, which are $O\left(0, 0\right)$ and $C \left(1, 1\right)$, and one saddle point, $E\left({x}^{*}, {y}^{*}\right)$. That is, both elderly social care organizations and health care organizations select the nonparticipation strategy or participation strategy. When the initial state of the system is in region *AOBE*, the system converges to $O\left(0, 0\right)$. That is, both elderly social care organizations and health care organizations select the nonparticipation strategy. When the initial state of the system is in region *ACBE*, the system converges to $C \left(1, 1\right)$. That is, both elderly social care organizations and health care organizations select the participation strategy. Therefore, the greater the area of region *ACBE* is, the greater the probability that the system will evolve to $C \left(1, 1\right)$. The area of region *ACBE* is expressed by Eq. (13):

13 $${S}_{\mathrm{ACBE}}=\frac{1}{2}\left[1\times \left(1-\frac{{C}_{2}-{R}_{2}-{S}_{1}^{^{\prime}}}{{R}_{1}-{R}_{2}+{P}_{1}-{S}_{1}^{^{\prime}}+{C}_{2}-{C}_{1}}\right)\right]+\frac{1}{2}\left[1\times \left(1-\frac{{P}_{2}-\left({U}_{2}+{S}_{2}\right)}{{U}_{1}-{U}_{2}+{P}_{2}-{P}_{1}+{S}_{1}-{S}_{2}}\right)\right]$$

Based on Eq. (13), the influence of each parameter on the evolutionary phase is shown in Table 5. We know the following:

**Table 5:** The impact of the parameter changes on the system evolution result

	$\frac{d{S}_{\mathrm{ACBE}}}{d{R}_{1}}$	$\frac{d{S}_{\mathrm{ACBE}}}{d{R}_{2}}$	$\frac{d{S}_{\mathrm{ACBE}}}{d{P}_{1}}$	$\frac{d{S}_{\mathrm{ACBE}}}{d{P}_{2}}$	$\frac{d{S}_{\mathrm{ACBE}}}{d{C}_{1}}$	$\frac{d{S}_{\mathrm{ACBE}}}{d{C}_{2}}$	$\frac{d{S}_{\mathrm{ACBE}}}{d{U}_{1}}$	$\frac{d{S}_{\mathrm{ACBE}}}{d{U}_{2}}$	$\frac{d{S}_{\mathrm{ACBE}}}{d{S}_{1}}$	$\frac{d{S}_{\mathrm{ACBE}}}{d{S}_{1}^{^{\prime}}}$	$\frac{d{S}_{\mathrm{ACBE}}}{d{S}_{2}}$
Symbol	$+$	$+$	$\pm$	$-$	$-$	$-$	$+$	$+$	$+$	$+$	$+$

1. When other parameters remain unchanged, region *ACBE* is positively correlated with ${R}_{1}$, ${R}_{2}$, ${U}_{1}$, ${U}_{2}$, ${S}_{1}$, ${S}_{1}^{^{\prime}}$ and ${S}_{2}$. This result indicates that the additional benefits affect the probability of choosing the participation strategy, and the increase in government subsidies helps to promote the subject synergy of integrated health care and social care services.
2. When other parameters remain unchanged, region *ACBE* is negatively correlated with ${C}_{1}$, ${C}_{2}$ and ${P}_{2}$. This result indicates that reducing the costs of establishing health care organizations in elderly social care organizations or the costs of providing elderly social care services in health care organizations can help to promote the subject synergy of integrated health care and social care services.
3. The influence of ${P}_{1}$ on region *ACBE* is not determined. When the costs that elderly social care organizations pay to purchase health care services or pay for negotiation in the bilateral cooperation type are too high, elderly social care organizations tends to withdraw from the cooperation. If the costs that elderly social care organizations pay to purchase health care services or pay for negotiation in the bilateral cooperation type are too low, health care organizations may withdraw from the cooperation due to the reduction in profit.

Scenario 2: When ${U}_{2}+{S}_{2}<{P}_{2}$ and ${R}_{2}+{S}_{1}^{^{\prime}}>{C}_{2}$, this means that in the elderly social care organization-led type, the additional benefits of elderly social care organizations are less than the costs for elderly social care organizations to provide extra health services. In the health care organization-led type, health care organizations provide extra elderly social care services, and the additional benefits and government subsidies for self-operated elderly care beds are more than the additional operating costs. The evolutionary phase diagram is shown in Fig. 1(b). There is one ESS, $C \left(1, 1\right)$, and two saddle points, which are $A\left(0, 1\right)$ and $B\left(1, 0\right)$. That is, both elderly social care organizations and health care organizations tend to choose the participation strategy.

Scenario 3: When ${U}_{2}+{S}_{2}>{P}_{2}$ and ${R}_{2}+{S}_{1}^{^{\prime}}<{C}_{2}$, this means that in the elderly social care organization-led type, the additional benefits of elderly social care organizations are more than the costs for elderly social care organizations to provide extra health services. In the health care organization-led type, health care organizations provide extra elderly social care services, and the additional benefits and government subsidies for self-operated elderly care beds are less than the additional operating costs. The evolutionary phase diagram is shown in Fig. 1(c). There is one ESS, $C \left(1, 1\right)$, and two saddle points, which are $O\left(0, 0\right)$ and $B\left(1, 0\right)$. That is, both elderly social care organizations and health care organizations tend to choose the participation strategy.

Scenario 4: When ${U}_{2}+{S}_{2}>{P}_{2}$ and ${R}_{2}+{S}_{1}^{^{\prime}}>{C}_{2}$, this means that in the elderly social care organization-led type, the additional benefits of elderly social care organizations are more than the costs for elderly social care organizations to provide extra health care services. In the health care organization-led type, health care organizations provide extra elderly social care services, and the additional benefits and government subsidies for self-operated elderly care beds are more than the additional operating costs. The evolutionary phase diagram is shown in Fig. 1(d). There is one ESS, $C \left(1, 1\right)$ and two saddle points, which are $O\left(0, 0\right)$ and $A\left(0, 1\right)$. That is, both elderly social care organizations and health care organizations tend to choose the participation strategy.

## Analysis of Numerical Simulation

To demonstrate the effectiveness of our model and better understand how the variables influence the evolutionary stable state of the system, a numerical simulation is conducted to analyse scenario 1 by using MATLAB software. In this scenario, the stability of the system is influenced by the initial strategy and the dynamic changes in the relevant parameters.

### The Impact of the Initial Strategy on the Evolutionary Results

The parameters are set as follows: ${U}_{1}=1$, ${U}_{2}=1.2$, ${R}_{1}=2.2$, ${R}_{2}=2.42$, ${S}_{1}=1.4$, ${S}_{1}^{^{\prime}}=1.59$, ${S}_{2}=1.6$, ${C}_{1}=4$, ${C}_{2}=4.21$, ${P}_{1}=2.23$, and ${P}_{2}=3$. It can be seen that these parameter values meet the condition in scenario 1. Thus, ${U}_{1}+{S}_{1}>{P}_{1}$ and ${R}_{1}+{P}_{1}>{C}_{1}$; additionally, ${U}_{2}+{S}_{2}<{P}_{2}$ and ${R}_{2}+{S}_{1}^{^{\prime}}<{C}_{2}$. With these values unchanged, we observe a change in the ESS by changing the values of the initial strategy. The initial probabilities are divided into two groups to demonstrate their impacts on the evolutionary results [46, 47].

**Fig. 2:**
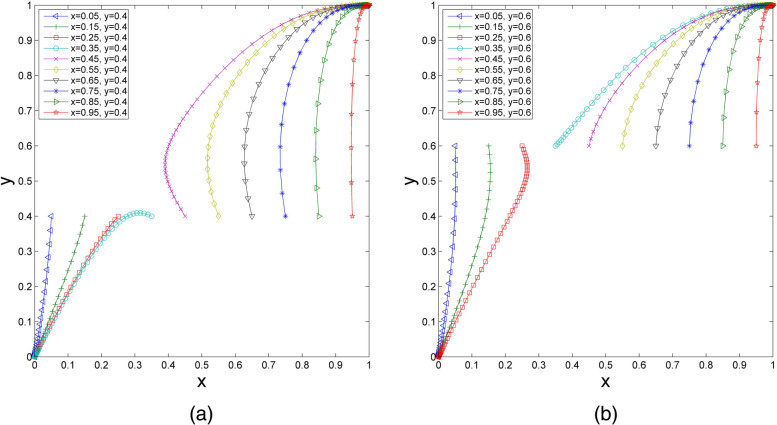
The impact of changes in $x$ on the evolutionary results. (**a**) $y$=0.4, wherein $x$=0.05, 0.15, 0.25, 0.35, 0.45, 0.55, 0.65, 0.75, 0.85, 0.95; (**b**)$y$=0.6, wherein $x$=0.05, 0.15, 0.25, 0.35, 0.45, 0.55, 0.65, 0.75, 0.85, 0.95

**Fig. 3:**
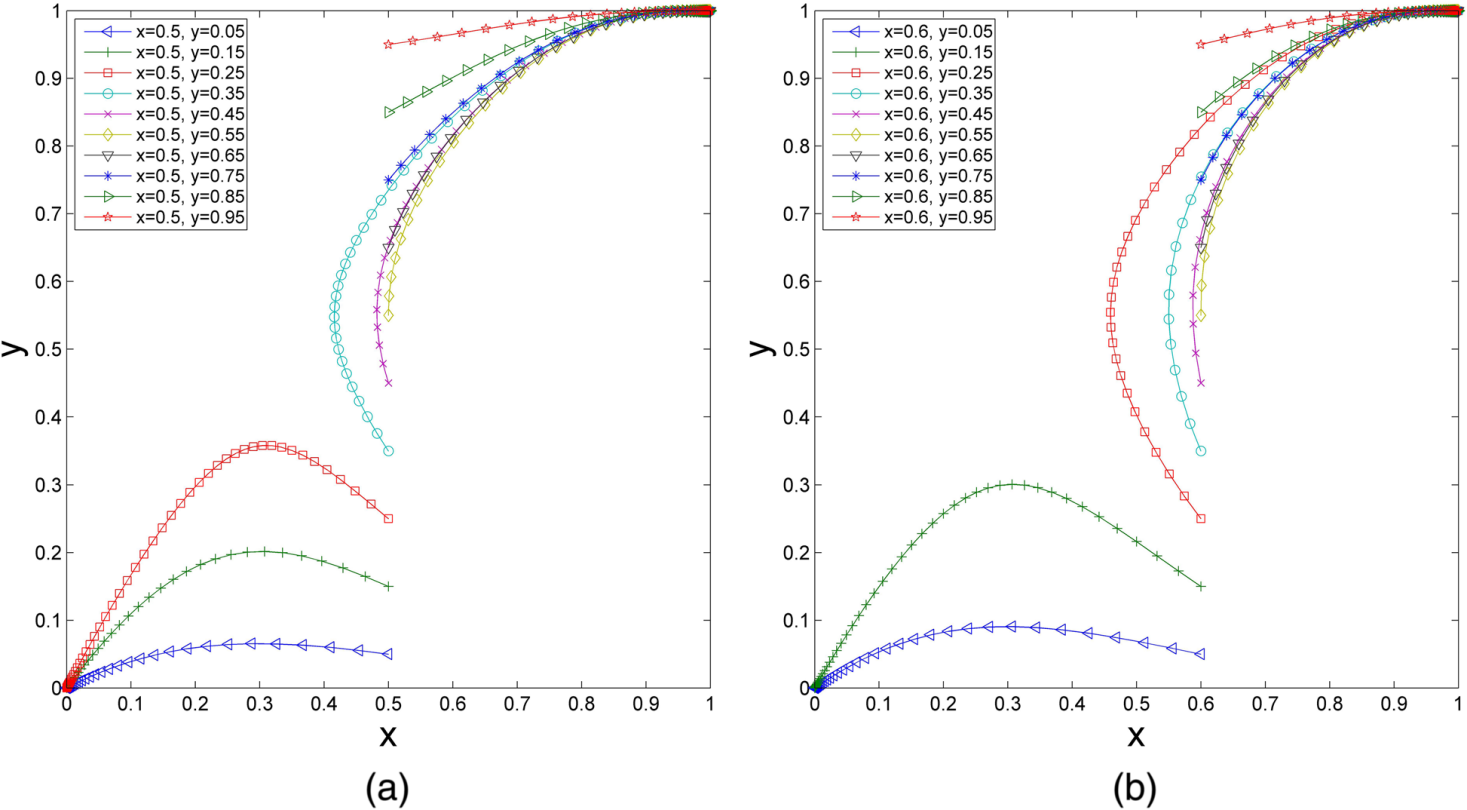
The impact of changes in $y$ on the evolutionary results. (**a**) $x$=0.5, wherein $y$=0.05, 0.15, 0.25, 0.35, 0.45, 0.55, 0.65, 0.75, 0.85, 0.95; (**b**)$x$=0.6, wherein $y$=0.05, 0.15, 0.25, 0.35, 0.45, 0.55, 0.65, 0.75, 0.85, 0.95

- (1) We set the initial probability of health care organizations choosing participation ($y$) as 0.4, and $x$ increases successively from 0.05 to 0.95. As shown in Fig. 2(a), when the value of $x$ increases to a certain extent, the ESS of the evolutionary system changes from $O\left(0, 0\right)$ to $C\left(1, 1\right)$. Similarly, assuming that the initial probability of health care organizations choosing participation is $y=0.6$, when $x$ increases successively from 0.05 to 0.95, the results are as shown in Fig. 2(b). With the value of $x$ increasing to a certain extent, the ESS of the evolutionary system changes from $O\left(0, 0\right)$ to $C\left(1, 1\right)$. This finding illustrates that under the same initial strategy of health care organizations, if the initial proportion of elderly social care organizations choosing participation is high enough, health care organizations ultimately tend to choose the participation strategy. In addition, with the increase in the initial probability of elderly social care organizations choosing participation, the speed of the system reaching ESS is improved. Comparing Fig. 2(a) and Fig. 2(b), with the increase in the initial probability of health care organizations choosing participation, the system evolves to $C\left(1, 1\right)$ faster.
- (2) When the initial probability of elderly social care organizations choosing participation $x=0.5$ is fixed and $y$ increases successively from 0.05 to 0.95, the changes are as shown in Fig. 3(a). If the value of $y$ increases to a certain extent, the ESS of the evolutionary system changes from $O\left(0, 0\right)$ to $C\left(1, 1\right)$. Similarly, when we set the initial probability of elderly social care organizations choosing participation $x=0.6$ and $y$ increases successively from 0.05 to 0.95, it can be seen in Fig. 3(b) that the ESS also changes from $O\left(0, 0\right)$ to $C\left(1, 1\right)$ if the value of $y$ increases to a certain extent. Under the same initial strategy of elderly social care organizations, when the initial probability of health care organizations choosing participation is high enough, the probability of elderly social care organizations choosing participation will stabilize at 1. Moreover, with the increase in the initial probability of health care organizations choosing the participation strategy, the evolutionary speed of the system accelerates. Comparing Fig. 3(a) and Fig. 3(b), if the initial probability of elderly social care organizations choosing participation increases, the system evolves to $C\left(1, 1\right)$ faster.

It can be concluded from the above analysis that the initial strategy will affect the replication dynamic system and evolutionary results of scenario 1. If the initial probabilities of health care organizations and elderly social care organizations choosing participation are high, they will be stable in synergizing with each other to provide integrated health care and elderly social care services. In contrast, if the initial probabilities of health care organizations and elderly social care organizations choosing participation are low, the system will evolve to $O\left(0, 0\right)$, which means neither of them will provide additional services. Both the increase in the probability of health care organizations choosing participation and the increase in the probability of elderly social care organizations choosing participation will speed up the evolution of the system to the stable point $C\left(1, 1\right)$, which reflects that the behavioural decisions on participation between health care organizations and elderly social care organizations influence each other.

### The Impact of Benefits on the Evolutionary Results

**Fig.4:**
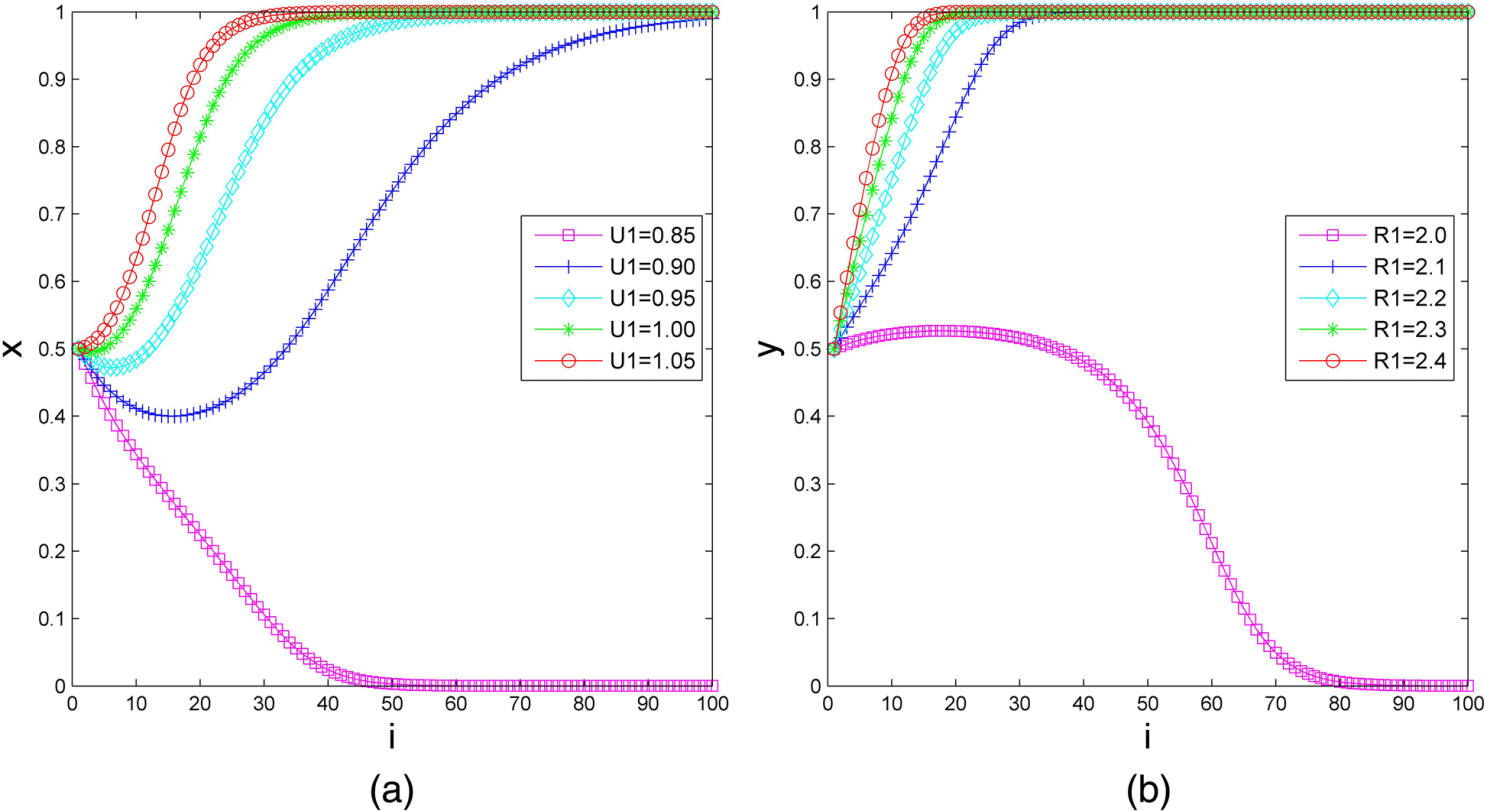
The impact of additional benefits on the evolutionary results. (**a**) For elderly social care organizations; (**b**) for health care organizations

Assuming that the initial probabilities $x=y=0.5$, first, we set the additional benefit of elderly social care organizations from the provision of health care services in the bilateral cooperation type (${U}_{1}$) as 0.85, 0.90, 0.95, 1.00 and 1.05, and we set the other parameters as ${U}_{2}=1.2$, ${R}_{1}=2.2$, ${R}_{2}=2.42$, ${S}_{1}=1.4$, ${S}_{1}^{^{\prime}}=1.59$, ${S}_{2}=1.6$, ${C}_{1}=4$, ${C}_{2}=4.21$, ${P}_{1}=2.23$, and ${P}_{2}=3$. The impact of ${U}_{1}$ on the evolutionary results is demonstrated in Fig. 4(a). It can be seen that the strategy of elderly social care organizations transforms from nonparticipation to participation when ${U}_{1}$ increases from 0.85 to 0.90. With the increase in the additional benefits, the system’s evolution step shortens; notably, the system’s evolution step reduces from 100 to 30 when ${U}_{1}$ increases from 0.90 to 1.05. Furthermore, when ${U}_{1}=$ 0.90 and 0.95, the probability of elderly social care organizations choosing participation first decreases and then increases. This finding indicates that if the additional benefits are not high enough, the willingness to cooperate may decrease in a short time, but it will eventually stabilize in the participation strategy.

Next, we set the additional income of health care organizations from providing elderly social care services in the bilateral cooperation type ${R}_{1}$ as 2.0, 2.1, 2.2, 2.3 and 2.4, and we set the other parameters as ${R}_{2}=2.42$, ${U}_{1}=1$, ${U}_{2}=1.2$, ${S}_{1}=1.4$, ${S}_{1}^{^{\prime}}=1.59$, ${S}_{2}=1.6$, ${C}_{1}=4$, ${C}_{2}=4.21$, ${P}_{1}=2.23$, and ${P}_{2}=3$. The impact of ${R}_{1}$ on the evolutionary results is demonstrated in Fig. 4(b). From Fig. 4(b), we see that the strategy of health care organization is nonparticipation when ${R}_{1}=2.0$. With the other parameters unchanged, when the value of ${R}_{1}$ increases from 2.1 to 2.4, the strategy of health care organizations is participation, and the evolution step of the system shortens from 40 to 20.

The above observation elaborates that the additional benefits have an incentive effect on elderly social care organizations and health care organizations, which encourages them to synergize to provide integrated health care and elderly social care services.

### The Impact of Costs on the Evolutionary Results

**Fig.5:**
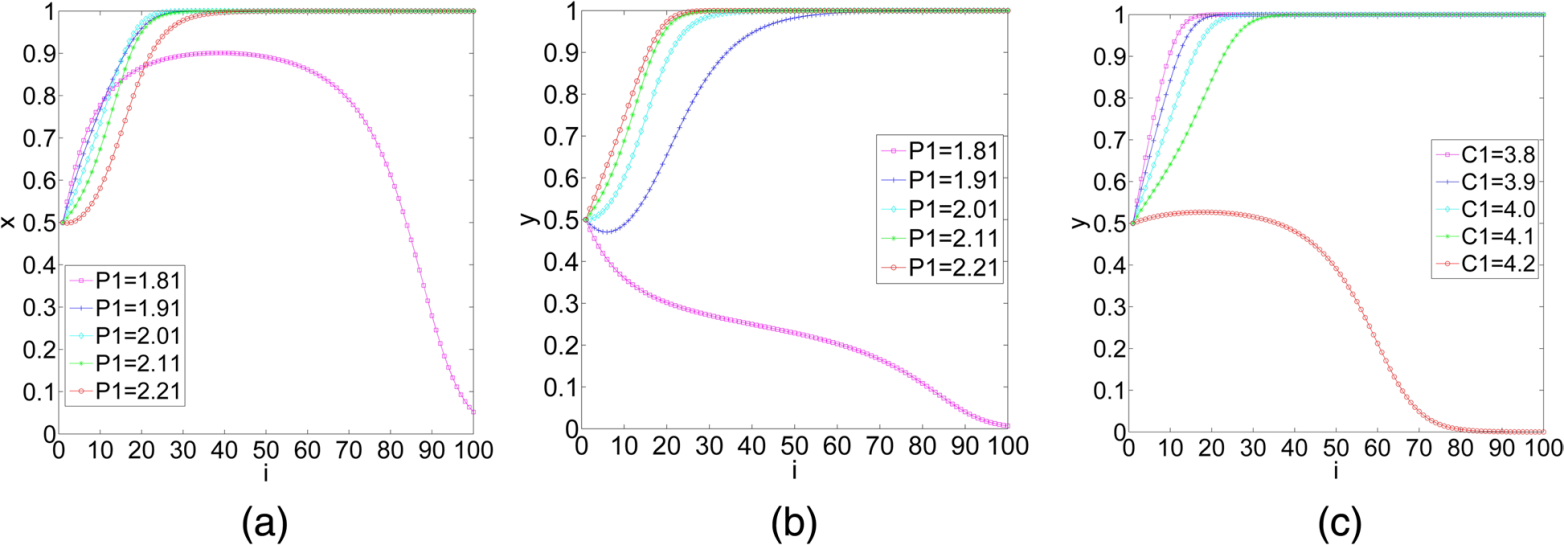
The impact of additional costs on the evolutionary results. (**a**) (**b**) The impact of the costs that elderly social care organizations pay to purchase health care services or pay for negotiation in the bilateral cooperation type on the evolutionary results, for elderly social care organizations and health care organizations, respectively; (**c**) the impact of the additional operating costs of health care organizations in the bilateral cooperation type on the evolutionary results

(1) We set the costs that elderly social care organizations pay to purchase health care services or pay for negotiation in the bilateral cooperation type (${P}_{1}$) as 1.81, 1.91, 2.01, 2.11 and 2.21, and we set the other parameters ${P}_{2}=3$, ${R}_{1}=2.2$, ${R}_{2}=2.42$,${U}_{1}=1$, ${U}_{2}=1.2$, ${S}_{1}=1.4$, ${S}_{1}^{^{\prime}}=1.59$, ${S}_{2}=1.6$, ${C}_{1}=4$, and ${C}_{2}=4.21$. Assume that the initial probability of elderly social care organizations and health care organizations choosing participation is $x=0.5$ and $y=0.5$, respectively. The impact of ${P}_{1}$ on the evolutionary results is shown in Fig. 5(a) and Fig. 5(b). In Fig. 5(a), when ${P}_{1}=1.81$, the rate at which elderly social care organizations evolve to the participation strategy increases rapidly in the early stage of evolution. After a period, the probability of elderly social care organizations evolving to participation declines quickly. From Figs. 5(a) and 5(b), we observe that the increase in the costs that elderly social care organizations pay to purchase health care services or pay for negotiation in the bilateral cooperation type can reduce the cooperation willingness of elderly social care organizations but increase the cooperation willingness of health care organizations in the early stage of evolution. In the middle and late stages of evolution, ${P}_{1}$ has the opposite effects on the decision-making of elderly social care organizations and health care organizations, and one’s selection affects the other’s selection. Therefore, with the increase in ${P}_{1}$, the final result of system evolution cannot be determined.

The numerical simulation verifies the above calculation results that the influence direction of ${P}_{1}$ on the evolutionary path of the system is uncertain.

(2) We set the additional operating costs of health care organizations in the bilateral cooperation type (${C}_{1}$) as 3.8, 3.9, 4.0, 4.1 and 4.2, and we set the other parameters as ${C}_{2}=4.21$, ${P}_{1}=2.23$, ${P}_{2}=3$, ${R}_{1}=2.2$, ${R}_{2}=2.42$,${U}_{1}=1$, ${U}_{2}=1.2$, ${S}_{1}=1.4$, ${S}_{1}^{^{\prime}}=1.59$, and ${S}_{2}=1.6$. Assume that the initial probability of elderly social care organizations and health care organizations choosing participation is $x=0.5$ and $y=0.5$, respectively. The impact of ${C}_{1}$ on the evolutionary results is shown in Fig. 5(c). We observe that health care organizations choose the participation strategy, and the evolutionary step of the system is extended from 20 to 40. When ${C}_{1}$ increases from 3.8 to 4.1, health care organizations change to choose the nonparticipation strategy when ${C}_{1}$ continues to increase to 4.2.

Therefore, we know that reducing the additional costs of health care organizations can effectively promote the synergy between health care organizations and elderly social care organizations.

### The Impact of Subsidies on the Evolutionary Results

**Fig.6:**
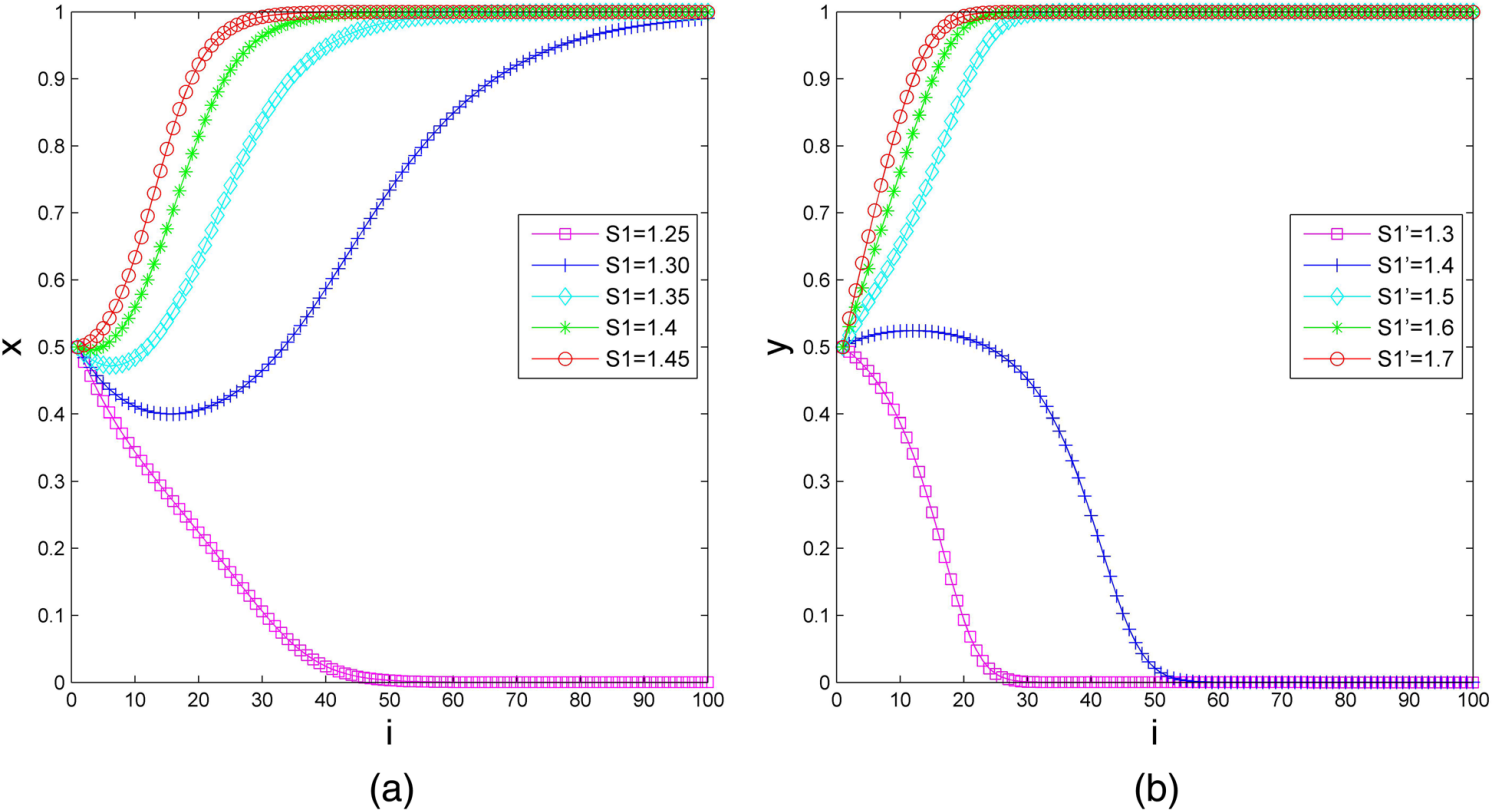
The impact of subsidies on the evolutionary results. (**a**) For elderly social care organizations; (**b**) for health care organizations

The government subsidy to elderly social care organizations in the bilateral cooperation type (${S}_{1}$) is set as 1.25, 1.30, 1.35, 1.40, and 1.45, and the other parameters are set as follows: ${S}_{1}^{^{\prime}}=1.59$, ${S}_{2}=1.6$, ${P}_{1}=2.23$, ${P}_{2}=3$, ${R}_{1}=2.2$, ${R}_{2}=2.42$, ${U}_{1}=1$, ${U}_{2}=1.2$, ${C}_{1}=4$, and ${C}_{2}=4.21$. We assume that the initial probability of elderly social care organizations and health care organizations choosing participation is $x=0.5$ and $y=0.5$, respectively. Figure 6(a) indicates the impact of subsidies to elderly social care organizations on the evolutionary results. It can be observed that elderly social care organizations change their strategy from nonparticipation to participation when ${S}_{1}$ increases from 1.25 to 1.3, and the stable strategy of health care organizations is participation when ${S}_{1}$ continues to increase from 1.3 to 1.45. More precisely, the length of time for health care organizations is greatly shortened.

Next, we set the government subsidy to health care organizations in the health care organization-led type $\left({S}_{1}^{^{\prime}}\right)$ as 1.3, 1.4, 1.5, 1.6 and 1.7, and we set the other parameters as ${S}_{1}=1.4$, ${S}_{2}=1.6$, ${P}_{1}=2.23$, ${P}_{2}=3$, ${R}_{1}=2.2$, ${R}_{2}=2.42$, ${U}_{1}=1$, ${U}_{2}=1.2$, ${C}_{1}=4$, and ${C}_{2}=4.21$. Then, we assume that the initial probability of elderly social care organizations and health care organizations choosing participation is $x=0.5$ and $y=0.5$, respectively. Figure 6(b) shows the numerical simulation of the impact of subsidies to health care organizations on the evolutionary results. If the government subsidy to health care organizations is small, health care organizations are inclined to choose the nonparticipation strategy. With other parameters unchanged, adjusting ${S}_{1}^{^{\prime}}$ to 1.5, health care organizations’ stable strategy changes to participation. As ${S}_{1}^{^{\prime}}$ increases from 1.5 to 1.7, the time for health care organizations to reach the stable state of participation is shortened.

Such numerical simulation illustrates that subsidy policy can effectively prompt the subject synergy between health care organizations and elderly social care organizations.

## Discussion

To the best of our knowledge, this is the first study to explore the cooperation strategies and organizational behaviours among health care and elderly social care organizations using the evolutionary game model. We draw the following conclusions: (1) The behavioural evolution of health care organizations and elderly social care organizations forms three types of integrated health care and social care services, which are the bilateral cooperation type, health care organization-led type and elderly social care organization-led type. The form in which evolution is stable mainly depends on the additional costs and benefits. (2) Increasing the additional benefits for cooperation and reducing the additional costs for cooperation can promote the willingness to synergize to provide integrated health care and elderly social care services. At the early stage of evolution, increasing the costs that elderly social care organizations pay to purchase health care services or pay for negotiation in the bilateral cooperation type could provide incentives for health care organizations to cooperate while reducing the cooperation preferences of elderly social care organizations. However, the long-term impact of the costs on the behavioural strategies for cooperation of the two players cannot be determined.

In the practice of integrated health care and social care services for elderly people in China, different sizes of health care organizations and elderly social care organizations face their own realistic dilemmas. For some smaller health care organizations, the health care level is relatively low, and there are many idle resources. Providing additional elderly social care services can improve the occupancy rate, increase the number of patients, boost the image of hospitals and obtain higher income with fewer costs. Therefore, such health care organizations are more inclined to participate in providing integrated health care and social care services for elderly people. However, due to the insufficient medical and staff resources in large general hospitals, providing elderly social care services leads to high labour costs but few benefits, so they lack the enthusiasm to provide integrated health care and elderly social care services. The cost of setting up health care organizations in elderly social care organizations is high due to the high entry threshold in health care fields. In such situations, the integrated health care and elderly social care services led by small and medium-sized elderly social care organizations can provide only basic nursing services, limited health care aid or referral services to primary care facilities and nursing homes, and only a small number of elderly social care organizations are able to provide all-inclusive care. However, the cost for such services constitutes a significant financial burden for most elderly individuals and decreases the number of elderly individuals who will enter this type of long-term care facility. On the other hand, a fee-for-service system and traditional health insurance in health care settings inevitably discourage less expensive technical services in community or long-term care facilities. In this regard, the conflicts between low benefits and high costs act as significant obstacles faced by local health care and elderly social care organizations while operating in a ‘disintegrated’ system. In other words, currently, integrated care is still weak in practice in China.

Community-based or family-based caregiving is a major component of long-term care within the Chinese context as well as from current public policy initiatives. As China moves into the next Five-Year National Development Plan, the challenge is to develop systematic care that provides integrated acute care, necessary long-term care and social support in the community that allows people to function in life activities. China is accelerating its ambitious plan to meet the increasing demands for integrated care; however, some lessons learned need to be adopted to consider a broader range of variations and complicated issues. The experience from integrated care in England showed that coordination across professional and organizational boundaries will remain the key challenge, and finding ways to support that may be more fruitful than designing complex integration initiatives [20]. Meanwhile, research on change within social and health services continues to indicate that deeply ingrained beliefs and practices and existing resource distribution, which often maintain the benefits and conditions of professional groups, inhibit the introduction of reform into complex systems [48]. Furthermore, shifting the focus from ‘integrated care’ to the ‘work required to integrate’ might provide a vehicle through which nascent partnerships can diagnose their problems and begin to design effective solutions [20].

Integrated care strategies might be most powerful where they become population oriented because policy-makers, professionals, and caregivers often find a mismatch between available service options and what service users truly need [49]. Research on the social service system reveals that the most effective way to promote system-level social change is to embed people who use social services at the centre of reform processes, both at the beginning and as they progress [48].

## Conclusions

Our study has shown that the behavioural decisions on participation between health care and elderly social care organizations influence each other. The commitment to integration and effective collaboration can be achieved by increasing the additional benefits and reducing the marginal costs. The findings help us to understand the potential and difficulties associated with cooperation in integrated health care service provision.

In the coming decades, the rapid growth of the elderly population in China will require both fiscal and substantive changes in integrated care that are responsive to the comprehensive need. Integrated care is characterized by complexity, including organizational, professional, cultural and technological types of cooperation. This study proposes a collaborative strategy among health care and elderly social care service providers. An evolutionary game model based on synergy theory is introduced. Furthermore, on the basis of stability analysis and parameters, sufficient conditions are studied, and the practical barriers to the strategy choice for an attractive solution in China are also described. Cooperation between actors can take place only in situations that contain a mixture of conflicting and changing interests. Addressing the complete identity of interests and emphasizing reciprocity at different levels of organizations from a synergy perspective are significant promoters for making integrated health care policy. Such cooperation decisions, however, transcend service providers, who should take into account the patient-oriented perspective. Involving service users, that is, elderly people, in the design of integrated care services and in decisions about the allocation of resources can help “unfreeze” the assumptions that providers make. It can also help providers reallocate resources to services that better meet service users’ needs.

## Data Availability

All data generated or analyzed during this study are included in this published article.
